# Vitamin D3 contribution to systemic and local control of *Salmonella* Typhimurium infection in an *in vivo* mouse model

**DOI:** 10.3389/fmolb.2026.1816390

**Published:** 2026-05-25

**Authors:** Michela Tarantino, Dorotea Ippolito, Giorgia Barbieri, Paola Petrucci, Alessia Tammaro, Roberta Tassinari, Francesca Maranghi, Emma Michetti, Serena Ammendola, Massimiliano Francia, Rosanna Adone, Paolo Pasquali, Barbara Chirullo

**Affiliations:** 1 Unit of Emerging Zoonoses, Department of Food Safety, Nutrition and Veterinary Public Health, Istituto Superiore di Sanità, Rome, Italy; 2 Unit of Gender-Specific Prevention and Health, Center for Gender-Specific Medicine, Istituto Superiore di Sanità, Rome, Italy; 3 Department of Biology, Tor Vergata University of Rome, Rome, Italy

**Keywords:** histopathology, immune response, *in vivo* model, *Salmonella* typhimurium, vitamin D3

## Abstract

**Introduction:**

Vitamin D serves as a critical modulator of innate and adaptive immune responses. Its active metabolite, 1,25-dihydroxyvitamin D3 (1,25(OH)_2_D_3_), exhibits potent antimicrobial activity in both humans and mice, positioning it as a promising adjunctive or alternative therapeutic for infectious diseases through immune modulation and clearance of pathogens. Among these, Salmonella enterica serovar Typhimurium, a leading cause of foodborne zoonotic infections, presents significant global public health burdens.

**Methods:**

Employing an in vivo murine model, we recapitulated the systemic and gastrointestinal features of S. Typhimurium-induced acute salmonellosis, which elicits a vigorous pro-inflammatory cytokine response that exacerbates pathogenesis while facilitating bacterial persistence. In this context, we evaluated the therapeutic potential of 1,25(OH)_2_D_3_ in an S. Typhimurium colitis model, examining its effects on infection severity, systemic immunity, and enteric immune dynamics. At three days post-infection, assessments encompassed bacterial colonization, flow cytometric analysis of peripheral blood leukocytes, and histopathological evaluation of tissues.

**Results:**

Our findings demonstrate that 1,25(OH)_2_D_3_ administration confers multifaceted protective effects, including reduced bacterial burden, attenuated histopathological inflammation, modulated immune cell profiles, and prolonged survival, thereby promoting infection resolution over progression.

**Discussion:**

These results underscore the immunomodulatory efficacy of vitamin D in combating S. Typhimurium infection and warrant further exploration for clinical translation.

## Graphical Abstract

**Figure:**
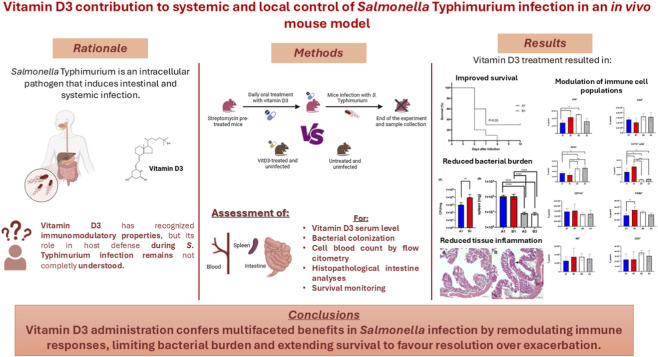

## 1 Introduction

Vitamin D is a group of fat-soluble prohormones comprising five distinct vitamins, with D2 (ergocalciferol) and D3 (cholecalciferol) being the most significant forms for human health (Sosa Henríquez and Gómez De Tejada Romero, 2020; Delrue and Speeckaert, 2023). Vitamin D2 is derived from plant sources, whereas vitamin D3 is found in animal-derived foods and it is primarily synthesized in vertebrates by the skin through exposure to sunlight (Kalpana et al., 2023; Van Den Heuvel et al., 2024). In recent years, lifestyle changes have led to an increasingly severe vitamin D deficiency across all population groups (Holick and Chen, 2008; Chang and Lee, 2019; Amrein et al., 2020; Canowitz et al., 2026). Beyond its well-established role in bone mineralization and health, and in regulating intestinal calcium and phosphorus absorption, vitamin D significantly influences both innate and adaptive immune responses. Growing scientific evidence links low vitamin D levels to increased cancer risk and mortality, chronic inflammatory and autoimmune diseases, cardiovascular conditions and higher susceptibility to microbial infections (Holick and Chen, 2008; Hewison, 2011; Canowitz et al., 2026).

Vitamin D’s active form, 1,25-dihydroxyvitamin D_3_ (1,25(OH)_2_D_3_), exerts biological activity by binding to the vitamin D receptor (VDR) and modulates the transcriptional program of VDR-target genes by interacting with the vitamin D response elements (VDRE) of target genes, such as involved in vitamin D metabolism and mineral balance (Sassi et al., 2018; Carlberg, 2022; Lebiedziński and Lisowska, 2023). VDR is expressed in most immune cells, including activated CD4^+^ and CD8^+^ T cells, B cells, neutrophils, antigen-presenting cells (APCs), and macrophages, as well as in approximately thirty tissues. This expression confirms the immunomodulatory effects of vitamin D and 1,25(OH)_2_D_3_, which directly regulate T-cell proliferation and cytokine secretion (Sassi et al., 2018; Martens et al., 2020).

*Salmonella enterica* serovar Typhimurium (*S.* Typhimurium) is a facultative intracellular bacterium and a leading cause of foodborne zoonotic infections, posing a major global public health burden via contaminated food and water (European Food Safety Authority EFSA, European Centre for Disease Prevention and Control ECDC, 2025). In immunocompetent individuals, it typically causes self-limiting gastroenteritis with symptoms like diarrhoea, abdominal pain, and vomiting, resolving in 4–7 days without treatment. Recognized as a priority antibiotic-resistant pathogen by the World Health Organization (WHO) and Centers for Disease Control and Prevention (CDC), *S*. Typhimurium includes multidrug-resistant (MDR) strains with extended-spectrum β-lactamases (ESBLs) or plasmid-mediated AmpC β-lactamases. These reduce the effectiveness of first-line drugs like fluoroquinolones and third-generation cephalosporins, leading to prolonged illness, higher treatment costs, increased transmission in healthcare and community settings, and elevated morbidity and mortality in humans and animals (Centers for Disease Control and Prevention U.S., 2019; WHO, 2024). As a facultative intracellular pathogen, *S.* Typhimurium employs sophisticated virulence mechanisms to invade and replicate within host cells, primarily non-phagocytic enterocytes and macrophages. Key virulence factors, encoded on *Salmonella* pathogenicity islands (SPIs), include two type III secretion systems (T3SS-1 and T3SS-2) that inject effector proteins to manipulate host cell signalling, cytoskeletal dynamics, and vesicular trafficking, thereby establishing protective *Salmonella*-containing vacuoles (SCVs) (Green et al., 2019; Galán, 2021). This intracellular niche enables evasion of humoral immunity, complement-mediated killing, and extracellular antimicrobials, while triggering host immune response involving innate pattern recognition receptors (e.g., TLR4, NLRs), cytokine storms (e.g., IL-1β, IL-18, TNF-α), and adaptive T-cell responses (Behnsen et al., 2015; Kurtz et al., 2017).

The interplay between bacterial persistence strategies and host clearance mechanisms underscores a critical “arms race” during infection. Enhanced understanding of *S.* Typhimurium pathogenesis, encompassing adhesion, invasion, intracellular survival, and immune modulation, offers opportunities to complement non-antibiotic interventions. Among them, novel microbial technologies are continuously evolving to combat bacterial infections and antimicrobial resistance, combining microbial biology with nanotechnology and engineering. The latter leverage synthetic biology to engineer microbes as targeted delivery vehicles for therapeutics or immunogens, inspired by probiotics’ natural colonization and tropism for specific tissues or pathogens (Palmer et al., 2018; Carolak et al., 2025; Kamble et al., 2025).

Epidemiological studies link vitamin D deficiency with increased susceptibility to enteric infections, while *in vitro* and *in vivo* evidence demonstrates that 1,25(OH)_2_D_3_ enhances antimicrobial peptide expression (e.g., cathelicidin LL-37 and β-defensins), promotes autophagy via ATG16L1 upregulation and augments macrophage phagolysosome maturation collectively altering the susceptibility of *S.* Typhimurium infection (Huang, 2016a; Huang and Huang, 2021). Such mechanisms could reduce reliance on conventional antibiotics, mitigate selection pressure for AR strains, and support host-directed therapies.

Acute salmonellosis can evoke an immune response characterized by an intensive production of pro-inflammatory cytokines, exploited for its own advantage, which is essential for favouring the dissemination of *S*. Typhimurium (Liu et al., 2012; Chirullo et al., 2015; Drumo et al., 2016). Within this frame, in the present study we investigate the immunomodulatory role of vitamin D3 during *S*. Typhimurium infection in murine models of both intestinal and systemic disease (Chirullo et al., 2015; Anderson and Kendall, 2017). We assessed how vitamin D3 influences host-pathogen interactions, aiming at its potential use as supportive adjuvant to antibiotic therapy or as a preventive strategy against antibiotic-resistant salmonellosis.

## 2 Materials and methods

### 2.1 *Salmonella* spp. culture

A wild-type strain of *S. enterica* serovar Typhimurium ATCC 14028 (hereafter named as STM) was used throughout the study, following standard protocols from previous works (Liu et al., 2012; Chirullo et al., 2015; Drumo et al., 2016). The cryopreserved strain was taken from the bacterial collection and was grown overnight at 37 °C to stationary phase in 100 mL of Brain Heart Infusion broth (Oxoid Ltd., UK) with agitation, harvested by centrifugation at 1,500 × *g* for 10 min, washed twice in ice-cold (±4 °C) phosphate buffer solution (PBS, P4417-50TAB, Sigma-Aldrich/Merk, Germany) and then diluted to a concentration equal to 0.2 mL × 10^4^ colony forming units (CFU).

### 2.2 *In vivo* studies and animal treatment

The animal study was performed in accordance with the Directive 2010/63/EU, the Italian Legislative Decree n. 26 of 4 March 2014 and the Organisation for Economic Co-operation and Development Principles of Good Laboratory Practice. The study protocol was approved by the Italian Ministry of Health (authorization n° 508/2018-PR). The animals were identified by applying subcutaneous transponders.

**FIGURE 1:**
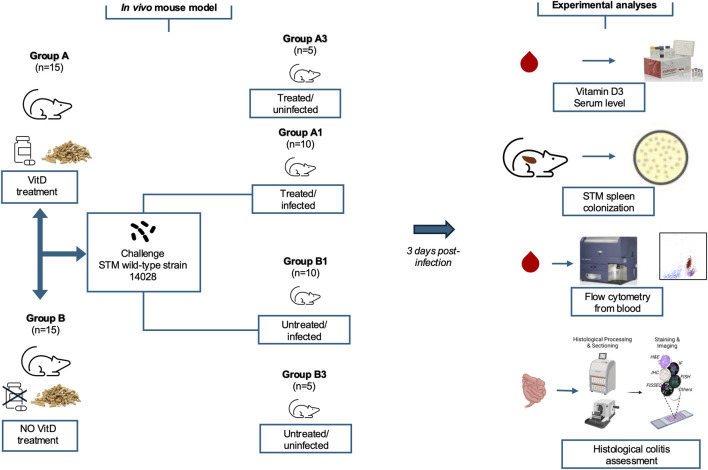
Experimental design and analyses. C57BL/6 mice were fed *ad libitum* for 7 weeks with (Group A) or without (Group B) supplementation of Vit D3. Ten mice from each group were orally infected with 10^4^ CFU/0.2 mL of the STM wild-type strain 14028 obtaining Group A1 and Group B1. Uninfected groups (A3 and B3, 5 mice per group) remained as controls. After 3 days post-infection, vitamin D serum level, bacterial colonization from spleen, complete blood counts by flow cytometry, and semi-quantitative histological colitis assessment were performed.

A *Salmonella* systemic and gastrointestinal *in vivo* model was established with 6-8-week-old female C57BL/6 mice. All mice were housed ventilated isolator cages (5 mice per cage) under standard laboratory conditions (22 °C ± 0.5 °C room temperature, 50%–60% relative humidity, 12 h of dark-light alternation with 12–14 air changes per hour) and regularly monitored weekly in term of body weight and food intake. After 7 days of acclimatising, thirty mice were divided into 2 groups: the first group (A = 15 mice) was fed with feed fortified with 75,000 I.U./kg of E 671-D3 (Altromin-R, Altromin International) *ad libitum* for 7 weeks (hereafter named as Vit D3); the 2nd group (B = 15 mice) was fed with standard diet, produced by the same company. After 7 weeks of Vit D3 or Mock treatment, 10 mice of group A (A1) and 10 mice of group B (B1) were treated with Streptomycin, 24 h before oral infection with STM (0.2 mL/mouse of 10^4^ CFU). The remaining mice received a dose of sterile PBS (P4417-50TAB, Sigma-Aldrich/Merk, Germany) via an oral feeding tube (gavage), serving as a control group (groups A3 and B3). Mice were, therefore, subdivided into four groups: 10 mice fed with Vit D3-fortified feed and inoculated with STM (A1); 10 mice inoculated with STM (B1), 5 control mice fed with Vit D3 fortified feed Vit D (A3); 5 negative control mice for STM and Vit D (B3), as described in Figure 1.

Blood samples were collected from the retro-orbital sinus from all mice. Three days post-infection, mice were euthanized, and intestinal tissue and spleen were collected for further analysis. Samples collection procedures are described below. Moreover, a satellite group of 10 additional mice per group (A1 and B1) was maintained and monitored for survival curve analysis. All the experiments were repeated twice, at least, with similar results.

The mice were euthanized in accordance with the Institutional Animal Care and Use Committee (IACUC) guidelines by cervical dislocation, following general anaesthesia induced by inhalation of a mixture of oxygen and 5% isoflurane in a sealed container, one mouse at a time. The depth of anaesthesia was confirmed by the absence of a response to a firm toe pinch or the loss of the righting reflex before performing cervical dislocation.

### 2.3 Assessment of vitamin D3 serum level

Vitamin D3 serum concentration was assessed at 6 weeks following the initiation of the Vitamin D3-enriched diet. Blood samples were collected from the retro-orbital sinus of all mice using non-coagulant tubes. Prior to sample collection, local anaesthesia was applied (Novasin 4%, one drop per eye). The blood samples were allowed to clot at room temperature for 30–60 min. The resulting serum samples were analysed using a specific immunoenzymatic assay with a commercially available ELISA kit (25-Hydroxy Vitamin D3 Competitive ELISA Kit, Invitrogen), following the manufacturer’s instructions. This method detects and quantifies the concentration of 25-dihydroxyvitamin D3 (25(OH_2_)D_3_, calcifediol) in serum, which is a prohormone produced by the liver by hydroxylation of vitamin D3 and is used to measure vitamin D status and detect vitamin D deficiency.

### 2.4 Cell blood count (CBC) by flow cytometer

Analysis of immune cells from 150 μL heparinised blood obtained from the retro-orbital sinus of the sedated mice was conducted by flow cytometry (MACSQuant analyzer 10 Flow Cytometer, Miltenyi Biotec), 3 days post-STM infection, at least two times.

The following flow cytometry protocol was used according to recent literature with some modifications (Arlt et al., 2024; Kamble et al., 2026):

- The erythrocytes were lysed using a lysis buffer (Red Blood Cell Lysis Buffer solution, 11814389001, Roche (100 mL) before staining.
- Cells were harvested and washed with PBS-5% FBS (FBS, GIBCO-BRL, USA) and stained with antibodies for 15 min in the dark at 4 °C.
- A master mix was prepared in advance, by dissolving 0.3 μg of each antibody in 90 μL of FACS buffer.
- The following antibodies were used: CD11b-VioBright FITC, mouse, Clone REA592; Anti-NK1.1-PE, mouse, Clone REA1162; CD4-PerCP-Vio700, mouse, Clone REA604; Anti-F4/80-PE-Vio770, mouse, Clone REA126; CD3-APC, mouse, Clone REA641; Anti-Ly-6G-APC-Vio770, mouse, Clone REA526; CD45-VioBlue, mouse; CD45R (B220)-VioGreen, mouse (Miltenyi Biotec s.r.l.).
- 100 μL of the antibody master mix was added to 100 μL of the blood cell suspension and the suspension was incubated for 15 min at 4 °C in the dark. Then, 1 mL of FACS buffer was added and the tubes were centrifuged for 5 min at 4 °C with 500 × *g*. Then cells were washed with PBS-5% FBS and fixed for 20 min at 4 °C with 1% paraformaldehyde (Formaldehyde solution about 37% EMPROVE® EVOLVE, Merck Life Science S.r.l.) in PBS.
- Cells were washed with PBS-5% FBS, the supernatant was discarded and the tubes were filled with 200 μL of FACS buffer. The samples were stored on ice in the dark until analysis, within 2 h.
- The acquisition samples were stopped at 50,000 events per sample; a gate was set within the lymphocyte-monocyte live populations using FSC/SSC strategy, singlet gating (FSC-A vs. FSC-H), then gate strategy was applied: CD45^+^, CD45^+^ CD3^−^, CD45^+^ CD3^+^ CD4^−^ CD56^−^, CD45^+^ CD3^+^ Cd4^+^, CD45^+^ B220^+^, CD45^+^ CD11b^+^ Ly6g^+^, CD45^+^ Cd11b^+^, CD45^+^ F4/80^+^.
- Compensation was carried out with anti-REA compensation beads (MACS® Comp Bead Kit, anti-REA, 130-104-693, Miltenyi Biotec s.r.l.) and the MacsQuant auto-compensation tool. The compensation results were controlled and manually modified with the help of single staining and unlabeled controls. Parent gating positive cells (%) were presented (percent events above fluorescence threshold set using negative control). A clearly shifted population within statistical differences between the treated-untreated groups were reported as results. Report mean ± SD from biological replicates. The flow cytometer analysis was performed using Flow Jo V10 program (FlowJo, LLC).

### 2.5 Evaluation of STM colonization

Three-days after infection the animals were euthanized. Following euthanasia, each mouse underwent a post-mortem examination to evaluate gross changes in the abdominal organs. Each organ was carefully excised and placed in a sterile Petri dish. Spleens were individually weighed. All tissues were homogenized in PBS and the number of viable STM was determined by plating serial dilutions on agar plates prepared using Brain Heart Infusion broth (Oxoid Ltd., UK) and agar (Oxoid Ltd., UK). Data is presented as per mg of tissue. *Salmonella* Typhimurium colonies were confirmed serologically as *Salmonella* by polyvalent agglutinating serum (*Salmonella* groups A-S, product r30858201, Remel Agglutinating Sera) based on the White-Kauffmann-Le Minor scheme (ISO 6579-3).

### 2.6 Post-mortem examination and histopathological assessment of colitis

The entire intestine was dissected and assessed for the presence of macroscopic haemorrhages. Mesenteric lymph nodes were excised individually and examined for signs of enlargement and lymphadenitis. Approximately 1 cm-long sections of the proximal colon were isolated. After a gentle wash-out of the luminal contents, each specimen was placed in a histology cassette and fixed in 10% neutral-buffered formalin. The sections were subsequently transferred to 70% ethanol for storage until paraffin embedding. Subsequently, colon samples were dehydrated in a graded series of alcohol baths and embedded in paraffin by the tissue processor (Shandon Excelsior ES, Thermo Scientific). Five-micron thick sections were cut by the Microm HM 325 (Thermo Scientific), stained with hematoxylin and eosin, and examined for signs of inflammation according to the criteria outlined by Walker et al. (2023). Slides were analysed under light microscopy using a Nikon Microphot FX microscope. In brief, four histological parameters were assessed in each colon section: mononuclear cell infiltration, neutrophilic inflammation, submucosal thickness/presence of oedema, and epithelial injury. These parameters were scored on a scale from 0 to 3 (absent to severe) and multiplied by an extent score based on the percentage of the section affected (ranging from 0 to 4).

### 2.7 Statistical analysis

For the *in vitro* and *ex vivo* assays, the distribution of each parameter was assessed for normality using the Shapiro-Wilk test and for homogeneity of variances using Bartlett’s test. Depending on the normality of the data, either parametric or non-parametric tests were applied. The statistical significance of differences in CFU splenic colonization between study groups was evaluated using Student’s t-test. Differences in serum vitamin D concentrations and immune cell subpopulation counts were analysed using ANOVA, followed by Tukey’s *post hoc* tests.

For histological scores, the effects of infection and treatment were assessed by the non-parametric Wilcoxon rank-sum (Mann-Whitney) test. Significant differences in histological parameters were then further tested using the Kruskal-Wallis test, with pairwise comparisons performed using Dunn’s *post hoc* method and a Bonferroni correction for multiple comparisons (Table 1).

**TABLE 1:** Results from Kruskal-Wallis test with Dunn’s *post hoc* comparisons and Bonferroni correction for multiple testing, comparing the medians of the histological parameters studied.

Histological item	A1 (n = 6)	A3 (n = 7)	B1 (n = 5)	B3 (n = 3)	Kruskall-Wallis test	
	Mean (±s.d.)	Mean (±s.d.)	Mean (±s.d.)	Mean (±s.d.)	Chi2 (3)	p-value
Mononuclear infiltration	10.83 (1.83)^a^	1.85 (0.377)^b^	12 (0)^a^	0 (0)^b^	18.503	<0.01
Neutrophilic inflammation	7.66 (4.08)^a^	0.28 (0.48)^b^	11.4 (1.34)^a^	0 (0)^b^	17.237	<0.01
Submucosal width	6.5 (1.91)^a^	1.66 (1.63)^b^	8.6 (3.5)^a^	0 (0)^b^	13.5	<0.01
Epithelial injury	1.5 (1.7)^a^	0.14 (0.37)^a,b^	7.2 (5.44)^a^	0 (0)^a,b^	7.190	0.02

Values with different superscript letters (a, b; P ≤ 0.05) denote significant differences between the experimental Groups.

Survival differences were analysed using mortality curves and assessed with the log-rank Mantel-Cox test. Statistical significance was set at *p* < 0.05. All analyses were conducted using STATA software (version 18.0).

## 3 Results

### 3.1 Vit D3 supplementation reduces the severity of STM infection and increases the survival rate

**FIGURE 2:**
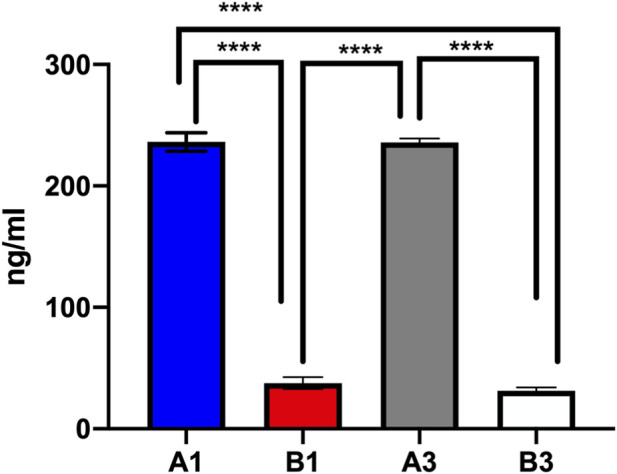
Vit D3 serum concentration confirmed the hight level of Vit D3 in the treated groups A1 and A3. Six weeks post-Vit D3 treatment, the Vit D serum concentration was significantly higher in both groups of Vit D-treated mice (A1-A3, 10 mice per group) compared to the untreated groups (B1-B3, 10 mice per group) (Kruskal-Wallis test, ****p = 0.0003).

The 25(OH)_2_D_3_ concentration were assessed at 6 weeks after the start of food fortified with vitamin D3, in all groups of mice, as control of the vitamin D3 serum concentration before the infection (Figure 2). As expected, the results indicate that after 6 weeks of treatment the Vit D3 serum concentration was significantly higher in both groups of treated mice (A1-A3) compared to the untreated groups (B1-B3).

**FIGURE 3:**
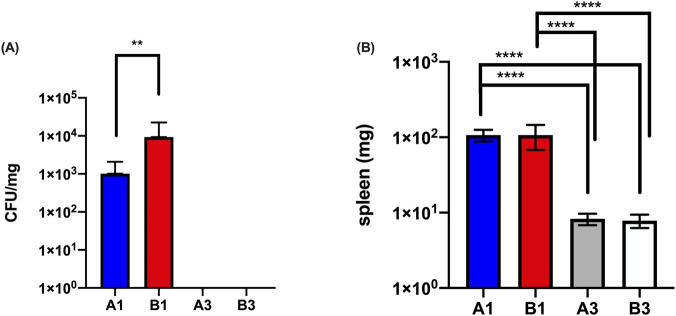
Vit D3 reduces the STM spleen colonization. **(A)** STM CFU splenic colonization, differences between A1 and B1 groups (with or without Vit D3, respectively). Intracellular colonization of STM in spleen cells at 3 days post-treatment (10 animals per group, t-test, *p* ≤ 0.005). No CFU were observed respectively in both A3 and B3, as uninfected groups. **(B)** Spleen weight measurements between the infected and uninfected groups, expressed in mg.

To evaluate the bacteria colonization, 3-days post-infection, mice were euthanized and spleen tissue samples were collected, weighted and homogenized. Group A1 (Vit D3-fed, STM-infected) exhibited significantly reduced STM colonization (*p* < 0.001, *t*-test) compared to Group B1 (standard diet, STM-infected), as verified by CFU counts and post-mortem necropsy. Approximately 1.02 × 10^3^ CFU/mL (2.037 CFU/mg) and 9.3 × 10^3^ CFU/mL (18.6 CFU/mg) were found in spleens of group A1 and B1, respectively (*t*-test, *p* < 0.005; Figure 3A). The weight of the spleens was similar between the two groups (Figure 3B). For further confirmation, STM colonies were confirmed serologically as *Salmonella* by polyvalent agglutinating serum (data not shown).

**FIGURE 4:**
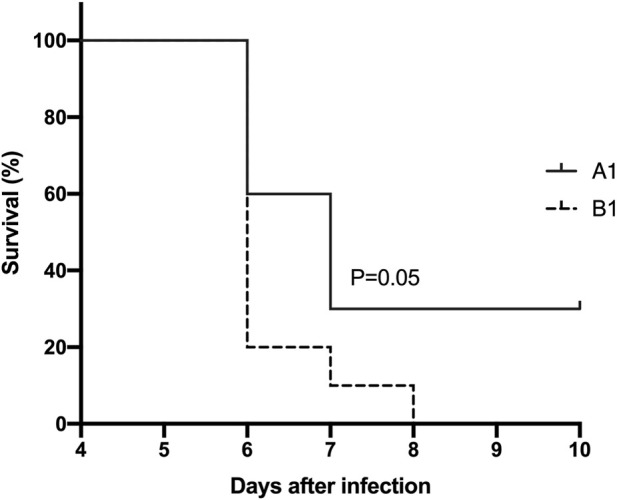
Vit D3 treatment increases the survival rate. Ten mice per groups (A1 and B1) was maintained and monitored for survival curve analysis: dashed line identifies the group the STM-infected mice (B1), solid line the group STM-infected and treated with Vit D3 (A1) (log-rank mantel-cox test, *p* = 0.05). The differences in survival rates demonstrates that the Vit D3 administration significantly increases the average life expectancy of the STM infected-animals compared to the untreated-control group (*p* = 0.05).

Moreover, other mice were kept evaluating the survival curve. The differences in survival rates demonstrates that the Vit D3 administration significantly increases the average life expectancy of the STM infected-animals compared to the untreated-control group (*p* = 0.05, Figure 4).

### 3.2 STM modify the subpopulation of immune cells Vit D3-treated mice cell

**FIGURE 5:**
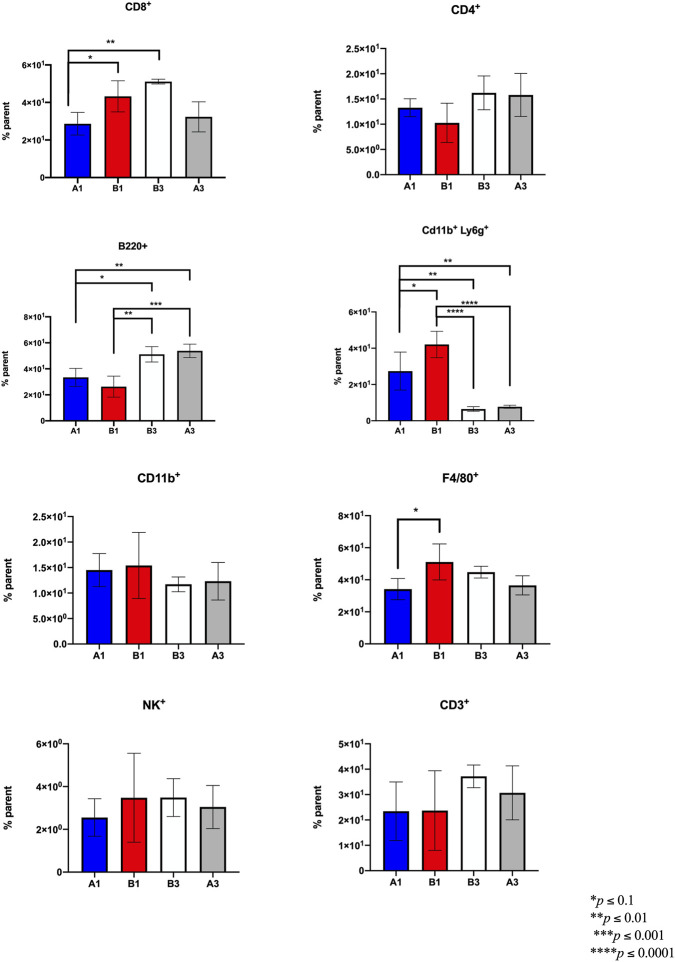
STM modify the subpopulation of immune cells in Vit D3 treated mice cell. Immune profile (cell blood count) by flow cytometry of A1, B1, A3, B3 groups. Flow-cytometry results of parent gating (Gated Population): CD45^+^, CD45^+^ CD3^−^, CD45^+^ CD3^+^ CD4^−^ CD56^−^, CD45^+^ CD3^+^ Cd4^+^, CD45^+^ B220^+^, CD11b^+^ Ly6g^+^, Cd11b^+^, F4/80^+^ (One-way Anova with Turkey’s multiple comparison test: *p ≤ 0.1, **p ≤ 0.01, ***p ≤ 0.001, ****p ≤ 0.0001).

To further evaluate the effects of Vit-D treatments, we analysed the dynamic movement and redistribution of the immune cells phenotyping, in particular on CD45^+^ CD3^+^ CD4^−^ T cells, CD45^+^ CD3^+^ CD4^+^ T cells, CD8 (CD45^+^ CD3^+^CD4^+^CD56^−^) T cells, macrophages (F4/80^+^) and neutrophils (CD11b^+^, Ly6g^+^), CD11b^+^ (monocytes), NK^+^, B (B220^+^) cells blood (Figure 5).

The results indicated that Vit D3 treatment induced a significant reduction of CD8^+^ T-cells, neutrophils, and macrophages (Figure 5) in STM infected mice (A1) versus uninfected mice (B1) (*p* < 0.01), while monocytes, CD4^+^ and NK^+^ cells have no significant differences between the groups, except a slight but no-significant increase in B lymphocyte. Moreover, the uninfected control groups A3 and B3 showed similar trend in almost all populations, indicating that Vit D3 in uninfected mice has no specific effects on the immune populations.

### 3.3 Histological colitis assessment supports the flow cytometry results

During the post-mortem examination, macroscopic signs of enteritis, including haemorrhagic inflammation and enlargement of the mesenteric lymph nodes, were observed in all STM-infected untreated mice (Group B1), while no signs of inflammation were detected in the other experimental groups (data not shown), displaying a clearly visible difference between the vitamin D3-treated and untreated groups upon the supervision and in the opinion of an experienced veterinarian pathologist.

**FIGURE 6:**
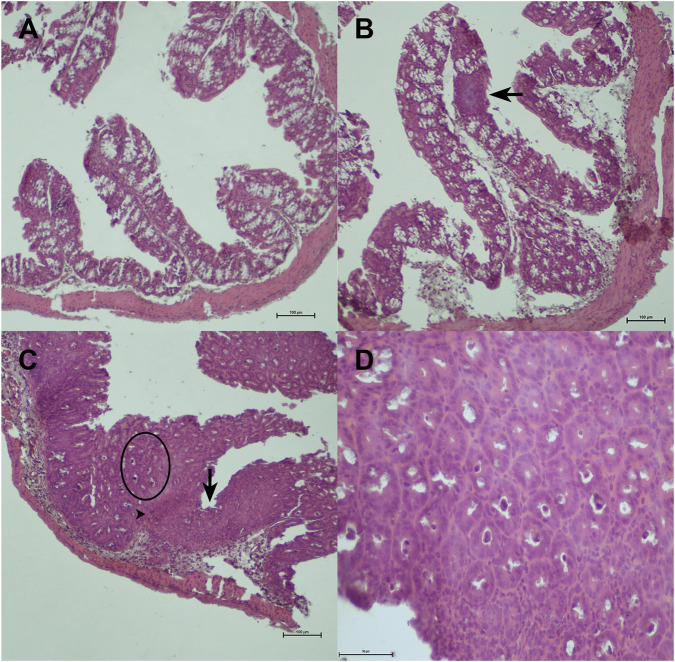
Histological assessment of colonic inflammation. Proximal colon sections, Hematoxylin/Eosin staining. **(A)** Group A3 (uninfected/untreated), 100×. Normal colon structure, no inflammation with normal crypt and goblet cells; **(B)** Group A1 (infected/treated), 100×. Moderate colonic inflammation, with focal infiltration of mononuclear cells and neutrophils (arrow) and severe oedema (asterisks); **(C)** Group B1 (infected/untreated), 100x. Severely inflamed colonic section showing alterations in mucosal architecture and extensive epithelial erosion (arrow). Mucosal and submucosal thickening. The mucosal layer displays diffuse inflammatory infiltrate, crypt loss (arrowhead) and with cryptitis (circled). Presence of submucosal oedema. **(D)** Group B1 (infected/untreated, higher magnification), ×400. Severe diffuse inflammatory infiltrate of the mucosa, with cryptitis and crypt abscesses.

The analysis of the haematoxylin/eosin-stained sections showed that STM infection significantly affected the intestinal wall response (Figure 6). Specifically, mice from Groups A1 and B1 (6 and 5 sections analysed respectively, 11 samples in total) exhibited higher scores for mononuclear cell infiltration, neutrophilic inflammation, submucosal involvement, and epithelial damage compared to uninfected mice from Groups A3 and B3 (7 and 3 sections analysed respectively, 10 samples in total), with statistically significant differences (*p* < 0.01; Table 2). Notably, no non-specific effect was observed following vitamin D administration, as no significant differences were found between treated mice (Groups A1 and B3) and untreated mice (Groups B1 and A3; Table 2).

**TABLE 2:** Two-sample Wilcoxon rank-sum (Mann–Whitney) test- Effect of infection and treatment on the intestinal wall histological parameters assessed.

Histological parameter	Infection				Vit D3 treatment			
	Infected (Group A1-B1) * n = 11	Uninfected (Group A3-B3) n = 10	Mann–Whitney test		Treated (Group A1-A3) n = 13	Untreated (Group B1-B3) n = 8	Mann–Whitney test	
	Mean (±sd)	Mean (±sd)	z	*p*	Mean (±sd)	Mean (±sd)	Z	*p*
Mononuclear infiltration	11.36 (1.43)	1.3 (0.94)	−4.1	<0.01	6 (4.81)	7.5 (6.21)	0.23	0.81
Neutrophilic inflammation	9.36 (3.58)	0.2 (0.42)	−4.0	<0.01	3.69 (4.66)	7.12 (6)	1.1	0.3
Submucosal width	7.66 (3)	1.11 (1.53)	−3.5	<0.01	3.6 (3)	5.37 (5.2)	0.58	0.56
Epithelial injury	4.1 (4.72)	0.1 (0.31)	−2.7	<0.01	0.77 (1.36)	4.5 (5.55)	1.37	0.18

The non-parametric Kruskal-Wallis test revealed significant differences in the histological scores across the four experimental groups. All the parameters showed the highest scores in Group B1, followed by Group A1. In contrast, Groups A3 and B3 exhibited mild or no alterations in these parameters (see Table 1).

Post-hoc comparisons revealed that the median scores for both mononuclear and neutrophilic infiltration in treated (Group A1) and untreated (Group B1) STM-infected mice showed significantly higher scores compared to their uninfected counterparts (Groups A3 and B3, *p* ≤ 0.05). A similar trend was observed for submucosal involvement/oedema, although no significant difference was found between treatment groups (A1 vs. A3, *p* = 0.14). Regarding epithelial damage, STM-infected untreated animals (Group B1) exhibited significantly more severe damage compared to Groups A3 and B3 (*p* < 0.01), while no significant difference was observed in comparison to Group A1. Interestingly, vitamin D3 treatment modulated the severity of the inflammatory response in the colon following STM infection. The most pronounced effect was observed in the reduction of neutrophilic infiltration and protection against epithelial injury. The mean scores for these parameters in Group A1 (vitamin D3-treated) were 7.66 (±4.08) for neutrophilic infiltration and 1.5 (±1.7) for epithelial injury. These values were notably lower than those observed in Group B1 (untreated), where the mean scores were 11.4 (±1.34) for neutrophilic infiltration and 8.6 (±3.5) for epithelial injury, however not statistically significant.

**FIGURE 7:**
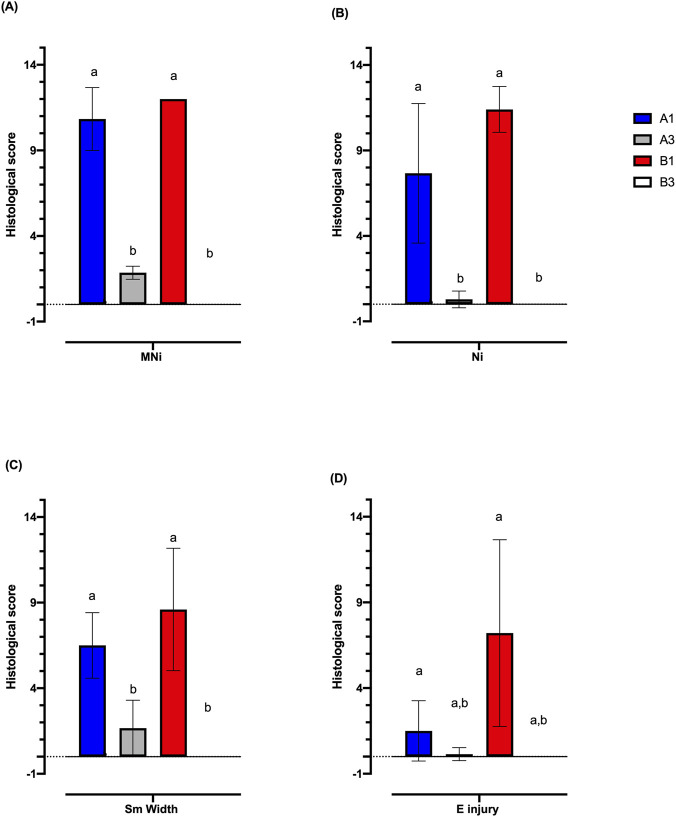
Histological assessment of colitis. Bar graph showing histological scores across groups, resulting from Kruskal-Wallis test with Dunn’s post-hoc comparisons and Bonferroni correction for multiple testing, comparing the medians of the histological parameters studied. Histological parameters, for each experimental group, were showed in: **(A)** MNi= Mononuclear infiltration; **(B)** Ni= Neutrophilic inflammation; **(C)** Sm Width= Submucosal width; **(D)** E injury= Epithelial injury. Values with different superscript letters (a, b; *p* ≤ 0.05) denote significant differences between the experimental Groups.

Mononuclear infiltration and submucosal involvement were also less severe in the vitamin D3-treated group (Group A1) compared to the untreated group (Group B1), although these differences were less pronounced. As shown in Figure 7 and Table 1, while the differences between Groups A1 and B1 were not statistically significant, a trend towards increasing values was observed for all parameters. The results are summarized in Figure 7 and Table 1.

## 4 Discussion

Over the past decade, the role of vitamin D3 in modulating immune responses to pathogens including *Salmonella* infection has garnered significant attention, supported by a growing body of *in vitro* and *in vivo* studies (Martin et al., 2015; Huang, 2016a; Huang and Huang, 2021; Shang et al., 2025; Caliman-Sturdza et al., 2026; Zeng et al., 2026). The active metabolite, 1,25-dihydroxyvitamin D_3_ (calcitriol), acts via the vitamin D receptor (VDR) expressed on immune cells to regulate both innate and adaptive immunity, highlighting the protective role of VDR signalling in host defence against pathogens (Daryabor et al., 2023). Indeed, studies report that VDR knockout-mice have increased bacterial burden and mortality following *Salmonella* infection and dysregulated pro-inflammatory responses, underlining the complex, context-dependent effects of vitamin D signalling (Wu et al., 2010; Chen et al., 2015).

In the present study, vitamin D3 administration reduces the burden of 14028 wild-type strain of *Salmonella* Typhimurium (STM) infection in a murine model, as evidenced by markedly reduced bacterial colonization, enhanced survival, and modulation of systemic and local inflammatory responses. At 3 days post-infection, Vit-D-treated mice (Group A1) displayed a ∼9-fold lower bacterial burden (2,037 CFU/mg) compared to untreated infected controls (Group B1). Consistent with this, survival curve analysis revealed a marked prolongation in median survival time in Vit D3-treated animals, reinforcing the potential role of vitamin D3 to reduce lethality in systemic salmonellosis. Such outcomes align with emerging evidence that Vit D3 optimizes innate antimicrobial pathways without compromising host viability (Huang and Huang, 2021; Guo et al., 2022). Indeed suboptimal vitamin D status (<30 ng/mL) may be associated with increased susceptibility to *Salmonella* gastroenteritis, while deficiency (<20 ng/mL) correlates with exacerbated inflammatory burden in hospitalized pediatric patients (Hung et al., 2026; Nicolae et al., 2026).

Flow cytometric analysis of peripheral blood further revealed selective immunomodulatory effects of vitamin D3, with significant reductions in circulating CD8^+^ T cells, neutrophils, and macrophages in infected mice receiving Vit D3 (A1) compared to untreated infected controls (B1), while CD4^+^ T cells, monocytes, NK cells, and B lymphocytes remained largely unaffected. These findings indicate a targeted dampening of pro-inflammatory effector populations, which are often exacerbated in acute bacterial sepsis and contribute to immunopathology. These infection-specific modulation could indicate that vitamin D tempers excessive pro-inflammatory effector responses, particularly those driven by neutrophils and macrophages, without inducing broad immunosuppression (Zhang et al., 2012; Charoenngam and Holick, 2020; Siddiqui et al., 2020; Daryabor et al., 2023). Uninfected controls (A3 and B3) immune profiles, regardless of Vit D3 treatment, confirm that these shifts are infection-dependent rather than constitutive effects of Vit D3. The selective reduction in inflammatory effectors, particularly neutrophils and macrophages, likely curtails immunopathology while preserving microbial containment, consistent with Vit-D’s role in enforcing homeostatic innate responses (Zhang et al., 2012; Daryabor et al., 2023). Therefore, these data suggest that Vit D3 has the capability to dampen the immune response versus *Salmonella* infection favouring the control of the infection. Moreover, the histopathological evaluation of the colon corroborated the systemic protective effects, revealing Vit D3-mediated attenuation of STM-induced enterocolitis. Macroscopic examination identified, indeed, haemorrhagic inflammation and mesenteric lymph node enlargement exclusively in untreated infected mice (B1), while such condition was absent in Vit D3-treated counterparts (A1). Quantitative scoring of hematoxylin-eosin-stained sections demonstrated elevated mononuclear and neutrophilic infiltration, submucosal oedema, and epithelial damage in both infected groups relative to uninfected controls. However, Vit D3 treatment substantially mitigated the neutrophilic infiltration and the epithelial injury. Although inter-group (A1 vs. B1) differences lacked statistical significance, a consistent trend toward reduced severity across all parameters supports Vit D3’s role in limiting neutrophil- and macrophage-driven tissue injury, hallmarks of *Salmonella* pathogenesis. This finding is aligned with the existing literature, which demonstrated that inflammation is strategically orchestrated by *Salmonella* to facilitate its survival and proliferation across diverse host environments (Patel and McCormick, 2014; Chirullo et al., 2015; Drumo et al., 2016; Zhao et al., 2025). Altogether these results support the flow cytometry results suggesting that the inflammation and the epithelial injury induced by STM infection was modulated by Vit D3 treatment.

Mechanistically, the protective effects of Vit D3 may stem from its well-established role in regulating innate and adaptative immunity, as also supported by extensive literature (Chun et al., 2014; Dimitrov and White, 2017; Charoenngam and Holick, 2020; Daryabor et al., 2023). Indeed, the active form of vitamin D, upregulates antimicrobial peptides (AMPs) such as mouse β-defensin-3 (mBD-3, the analogue of human β-defensin-2), and cathelicidin (in human) antimicrobial peptide expression thereby promoting bacterial killing (Liu et al., 2006; Huang and Huang, 2021). It also enhances pattern recognition and phagocytosis by upregulating Toll-like receptors (TLRs), NOD2, and CD14 in macrophages, which promotes chemotaxis and oxidative burst (Yuk et al., 2009; Chun et al., 2014; Wei and Christakos, 2015). Additionally, active vitamin D promotes autophagic clearance and reduces bacterial translocation during *Salmonella* infection through increased expression of proteins such as NOD2, ATG16L1, and LC3B, while modulating inflammatory responses to protect the host from excessive inflammation (Huang, 2016b). Pro-inflammatory cytokine mRNA levels in the cecum, including IL-1β, IL-6, TNF-α, and IL-8, are suppressed, whereas IL-10 is upregulated to prevent hyperinflammation (Dickie et al., 2010; Zhang et al., 2012; Chun et al., 2014; Bishop et al., 2021). Moreover, the vitamin D receptor (VDR) facilitates NLRC4 inflammasome assembly, thereby elevating IL-1β and caspase-1 levels to enhance pathogen clearance (Dimitrov and White, 2017).

(Liu et al., 2006; Yuk et al., 2009). In this context, the selective reduction in CD8^+^ T cells reflect Vit D3’s influence on T-cell trafficking or apoptosis in inflammatory contexts, potentially limiting cytotoxic immunopathology without compromising adaptive immunity. Furthermore, the elevated macrophage counts observed in the STM-infected untreated group (B1) relative to the vitamin D3-treated infected group (A1) directly correlate with the more severe tissue damage revealed by histological analysis, which was markedly pronounced in group B1 compared to group A1, which is as also supported by consistent literature (Zhang et al., 2012; Huang and Huang, 2021).

Therefore, vitamin D’s induction of AMPs underpins its antibiotic-like effects, while its broader immunomodulation integrates innate defences with adaptive responses for balanced immunity. This has profound implications for infections like *Salmonella* Typhimurium, where vitamin D reduces severity via AMPs and autophagy, and for human health, suggesting supplementation in deficient populations to curb antibiotic resistance and enhance vaccine responses. However, challenges include species-specific differences and optimal dosing. Ongoing research, including post-2020 studies on viral pandemics, continues to unravel these roles, advocating for vitamin D as a cost-effective adjunct in infectious and autoimmune disease management (Charoenngam and Holick, 2020; Sîrbe et al., 2022).

The present work presents, however, a sex bias limitation. Indeed, only female mice have been used. The main reasons rely on the fact that oestrogen exposure suppresses acute inflammatory responses and increases susceptibility to *Salmonella* in some contexts, while progesterone may enhance resistance by increasing peritoneal cell influx (Kita et al., 1989). Moreover, the inbred C57BL/6 mice have been chosen since they are highly susceptible strain to *S*. Typhimurium infections (Sebastiani et al., 2002; Walker et al., 2023). Therefore, the female C57BL/6 mice has been preferred to work in the worst-case scenario. The inclusion of both sexes and different mouse models are envisaged in future in order to perform a more comprehensive evaluation of the mechanisms.

In conclusion, Vit D3 administration confers multifaceted benefits in *Salmonella* infection by remodulating immune responses, limiting bacterial burden and extending survival to favour resolution over exacerbation. These findings strongly support further exploration of vitamin D3 as a safe, low-cost host-directed adjunctive therapy, particularly in vitamin D3-deficient individuals or in settings of emerging antibiotic resistance and warrant clinical trials combining vitamin D3 supplementation with standard antibiotics.

## Data Availability

The raw data supporting the conclusions of this article will be made available by the authors, without undue reservation.
